# Ischemic conditioning by short periods of reperfusion attenuates renal ischemia/reperfusion induced apoptosis and autophagy in the rat

**DOI:** 10.1186/1423-0127-16-19

**Published:** 2009-02-11

**Authors:** Hsing-Hui Wu, Tzu-Yu Hsiao, Chiang-Ting Chien, Ming-Kuen Lai

**Affiliations:** 1 Department of Medicine, Kuang-Tien General Hospital, Taichung, Taiwan; 2 Department of Otolaryngology, National Taiwan University Hospital and National Taiwan University College of Medicine, Taipei, Taiwan; 3 Department of Medical Research, National Taiwan University Hospital and National Taiwan University College of Medicine, Taipei, Taiwan; 4 Department of Medicine, National Taiwan University Hospital and National Taiwan University College of Medicine, Taipei, Taiwan

## Abstract

Prolonged ischemia amplified iscehemia/reperfusion (IR) induced renal apoptosis and autophagy. We hypothesize that ischemic conditioning (IC) by a briefly intermittent reperfusion during a prolonged ischemic phase may ameliorate IR induced renal dysfunction. We evaluated the antioxidant/oxidant mechanism, autophagy and apoptosis in the uninephrectomized Wistar rats subjected to sham control, 4 stages of 15-min IC (I15 × 4), 2 stages of 30-min IC (I30 × 2), and total 60-min ischema (I60) in the kidney followed by 4 or 24 hours of reperfusion. By use of ATP assay, monitoring O_2_^-. ^amounts, autophagy and apoptosis analysis of rat kidneys, I60 followed by 4 hours of reperfusion decreased renal ATP and enhanced reactive oxygen species (ROS) level and proapoptotic and autophagic mechanisms, including enhanced Bax/Bcl-2 ratio, cytochrome C release, active caspase 3, poly-(ADP-ribose)-polymerase (PARP) degradation fragments, microtubule-associated protein light chain 3 (LC3) and Beclin-1 expression and subsequently tubular apoptosis and autophagy associated with elevated blood urea nitrogen and creatinine level. I30 × 2, not I15 × 4 decreased ROS production and cytochrome C release, increased Manganese superoxide dismutase (MnSOD), Copper-Zn superoxide dismutase (CuZnSOD) and catalase expression and provided a more efficient protection than I60 against IR induced tubular apoptosis and autophagy and blood urea nitrogen and creatinine level. We conclude that 60-min renal ischemia enhanced renal tubular oxidative stress, proapoptosis and autophagy in the rat kidneys. Two stages of 30-min ischemia with 3-min reperfusion significantly preserved renal ATP content, increased antioxidant defense mechanisms and decreased ischemia/reperfusion enhanced renal tubular oxidative stress, cytosolic cytochrome C release, proapoptosis and autophagy in rat kidneys.

## Background

In ischemic diseases, hypoxic degree, severity and duration lead to cell death and determine tissue pathology [1]. However, hypoxic/ischemic tolerance in organs can be achieved by several types of preconditioning with sub-lethal stresses such as hypoxia [2], ischemia [3,4], and hyperthermia [5,6]. The renal tolerance also can be obtained with ischemic postconditioning [7] defined as rapid intermittent interruptions of blood flow in the early phase of reperfusion. The molecular mechanisms of several preconditioning and postconditioning methods possibly involve the initial release of adenosine, bradykinin, prostacyclin, nitric oxide, reactive oxygen species (ROS) as well as the Akt signaling transmitters [7-11]. A hypoxic/ischemic preconditioning method exhibits an " early mitochondrial-independent protective window" and "delayed mitochondrial -dependent protective window" to subsequently prolonged ischemic injury [12-18]. Ischemic conditioning (IC) with the interruption of ischemic stage by short periods of blood reperfusion or reflow may have a potential effect on ischemia/reperfusion (IR) induced renal dysfunction. However, as far we know, few literatures have been demonstrated.

Mitochondrial dysfunction critically contributes to apoptosis and autophagy [19,20]. Mitochondrial dysfunction led to apoptotic cell death (type I programmed cell death) via increases of cytoplasmic cytochrome C release and activation of several caspases [21,22]. Cytoplasmic cytochrome C release is promoted by Bax [23] and inhibited by Bcl-2/Bc-xL [20,21,24]. Autophagy is a type II programmed and caspase-independent cell death [25,26]. Increased ROS enhances Bax/Bcl-2, Bax/Bcl-xL ratio, active caspase 3 (CPP32) expression, and poly-(ADP-ribose)-polymerase (PARP) degradation fragments subsequently resulting in apoptotic cell death [20,21,27]. The increased ROS also upregulates the expression of the autophagy-promoting protein Beclin-1 and microtubule-associated protein light chain 3 (LC3) leading to autophagy [20,25,26]. Our recent data indicated IR injury increased renal tubular autophagy and apoptosis, which can be ameliorated by increased renal Bcl-2/Bcl-xL proteins [20]. We hypothesized that a reduction in ROS by appropriate IC may ameliorate renal dysfunction. Therefore, we evaluated different IC conditions on IR induced oxidative injury, apoptosis and autophagy in the rat kidney.

## Materials and methods

### Animals

Female Wistar rats (200–250 g) were housed at the Experimental Animal Center, National Taiwan University at a constant temperature and with a consistent light cycle (light from 07:00 to 18:00). The animal care and experimental protocols were in accordance with the guidelines of the National Science Council of the Republic of China (NSC 1997). The body weight of the animals was measured once a week. Food and water were provided ad libitum.

### Induction of ischemic renal failure

**Figure 1:**
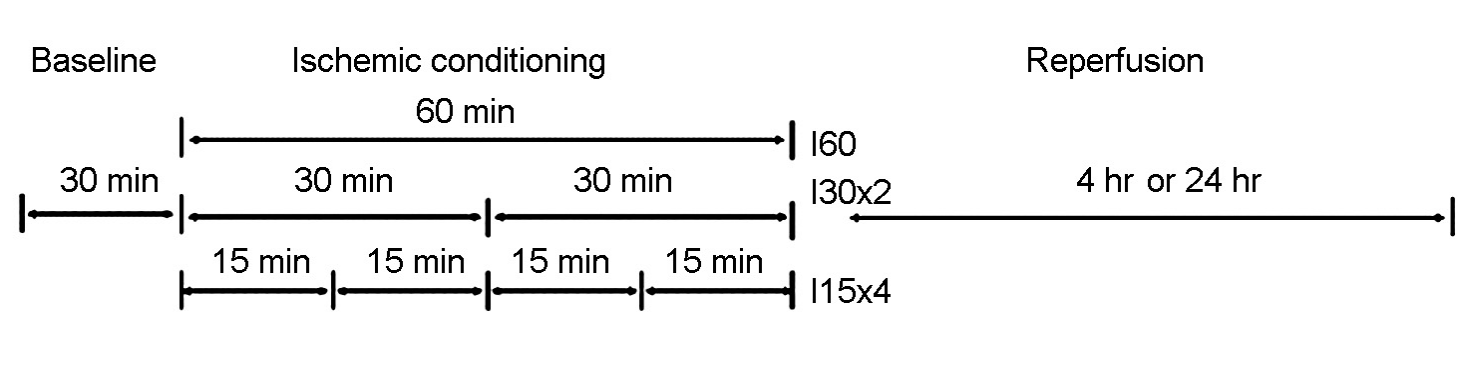
**Schedule for ischemic conditioning of the uninephrectomized rats in the experiment**. The left kidney was subjected to a 60-min ischemia (I60), one 3-min reperfusion interruption between 2 stages of 30-min ischemia (I30 × 2) or three 3-min reperfusion interruptions between 4 stages of 15-min ischemia (I15 × 4) followed by 4 hours (4 hr) or 24 hours (24 hr) of reperfusion in all the uninephrectomized rats. Each group contains 6 animals. All the animals were sacrificed after 4 hr or 24 hr of reperfusion.

We followed a surgical procedure as described in our previous study [20,21]. All the rats were performed a surgery with right kidney removal one week before the experiment. All the rats were anesthetized with sodium pentobarbital (40 mg/kg, i.p.). To induce ischemia in the left kidney, the left renal artery was clamped with a small vascular clamp for 60 min, two periods of 30 min ischemia interrupted by one stage of 3 min of reperfusion, or four periods of 15 min ischemia interrupted by three stages of 3 min of reperfusion. For simulating clinical practice to avoid ischemic injury, we selected 3 min of reperfusion period between two 30-min or four 15-min ischemic periods in this study. Sham-operated animals underwent similar operative procedures without occlusion of the left renal artery. The detailed protocol was indicated in Figure 1. Reperfusion was initiated by removal of the clamp for 4 or 24 hr. Following ischemia, the rats were allowed to recover for 4 or 24 hr of reperfusion. After different treatment of IR insult, arterial blood was collected to determine renal functions. Blood urea nitrogen (BUN) and plasma creatinine were analyzed using a commercial kit from Sigma (St Louis, MO, USA). Urine sodium concentration was collected with a PE-10 tube catheterization in the left ureter and was determined by flame photometry (Eppendorf, FCM6341, Hamburg, Germany). Urine sodium was expressed per gram of kidney weight. The rats were sacrificed with an overdose of anesthetics at the end of the experiment. Their kidneys were resected and divided into two parts. One part was stored in 10% neutral buffered formalin for *in situ *4-HNE, autophagy and apoptotic assay, and the other was quickly frozen in liquid nitrogen and stored at -70°C for adenosine triphosphate (ATP) determination and protein isolation.

### In vivo ROS recording

The ROS generation in response to ischemia/reperfusion injury was measured on the kidney surface by intra-renal arterial infusion of a super oxide anion probe, 2-Methyl-6-(4-methoxyphenyl)-3,7-dihydroimidazo- [1,2-a]-pyrazin-3-one-hydrochloride (MCLA) (0.2 mg/ml/h, TCI-Ace, Tokyo Kasei Kogyo Co. Ltd., Tokyo, Japan) and recorded by Chemiluminescence Analyzing System (CLD-110, Tohoku Electronic In. Co., Sendai, Japan) [21,27]. The real-time displayed chemiluminescence signal was recognized as ROS level on the kidney surface.

### NADPH oxidase assay

The NADPH oxidase capacity of kidney tissue samples was determined by using a lucigenin-amplified chemiluminescence assay, the most sensitive method of superoxide detection, which measures the NADPH oxidase-mediated ROS-generating system [24].

### Myeloperoxidase (MPO) assay

Renal tissue MPO activity, an enzyme marker of inflammation and neutrophil infiltration, was used as a marker for neutrophil content [28].

### ATP assay

After 4 hours of reperfusion, renal tissue ATP content was determined by using a chemiluminescence analysis [29]. The ATP content of renal homogenates was measured using a luciferin-luciferase assay kit according to the manufacturer's instructions (Roche, Penzberg, Germany). The method was used to determine the ATP dependency of the light-emitting luciferase-catalyzed oxidation of luciferin. At 0–4 hours, samples were diluted in a buffer containing 100 mM Tris and 4 mM EDTA (pH 7.75) and mixed immediately with equal amounts of the luciferase reagent. The light emitted from the luciferase was measured using a Chemiluminescence Analyzing System (CLD-110, Tohoku Electronic In. Co., Sendai, Japan), and the values were calibrated against a standard ATP curve.

### In situ detection of 4-HNE, autophagy and apoptosis

4-HNE, autophagy and apoptosis were used to detect *de novo *oxidative injury in the insulted kidney [20]. The value of brown deposits/total section area in the 4-HNE and autophagy was counted by Adobe Photoshop 7.0.1 image software analysis [21]. The method for the terminal deoxynucleotidyl transferase-mediated nick-end labeling method (TUNEL) was performed as previously described [27] to detect apoptosis *in situ*. Sections of the kidney were stained by methyl green and the TUNEL-avidin-biotin-complex method. Twenty high-power (×400) fields were randomly selected, and the value of apoptotic cells/(apoptotic cells and methyl green stained cells) was counted. The number of apoptotic cells was expressed per 100 of the tubular cells in each section.

### Immunoblot for Mn SOD, CuZn SOD, Catalase, Bax, Bcl-2, CPP32, PARP and LC3

The expression levels of antioxidant proteins including Mn SOD, CuZn SOD and catalase, apoptosis-related proteins including Bcl-2, Bax, caspase 3 (CPP32), and PARP and autophagy related proteins LC3 and Beclin-1 Western immunoblotting in kidney samples from rats with or without IR injury were detected as described previously [20,21,27]. Antibodies raised against polyclonal anti-Mn SOD (Stressgen Bioreagents Limited, Victoria, Canada), polyclonal rabbit anti-human CuZn SOD (Stress Marq Biosciences Inc., Victoria, Canada), catalase (Chemicon International Inc., Temecular, CA), Bax (Chemicon, Temecula, CA), Bcl-2 (Transduction, Bluegrass-Lexington, KY), the activation fragments of caspase 3 (CPP32/Yama/Apopain, Upstate Biotechnology, Lake Placid, NY), PARP degradation fragments (Promega, Madison, WI), LC3 (MLB), Beclin-1 (AnaSpec, Inc., San Jose, CA) and β-actin (Sigma, Saint Louis, MI) were used. All of these antibodies cross-react with the respective rat antigens. Ten μg of proteins were electrophoresed followed by immunoblot analysis.

### Statistical analysis

All values are expressed as mean ± SE. For comparisons of group data, one-way analysis of variance was applied first and if it is significant, the post-hoc test was conducted. P < 0.05 was considered to indicate statistical significance.

## Results

### Ischemic conditioning reduced ischemia- and reperfusion-induced kidney ROS levels

**Figure 2:**
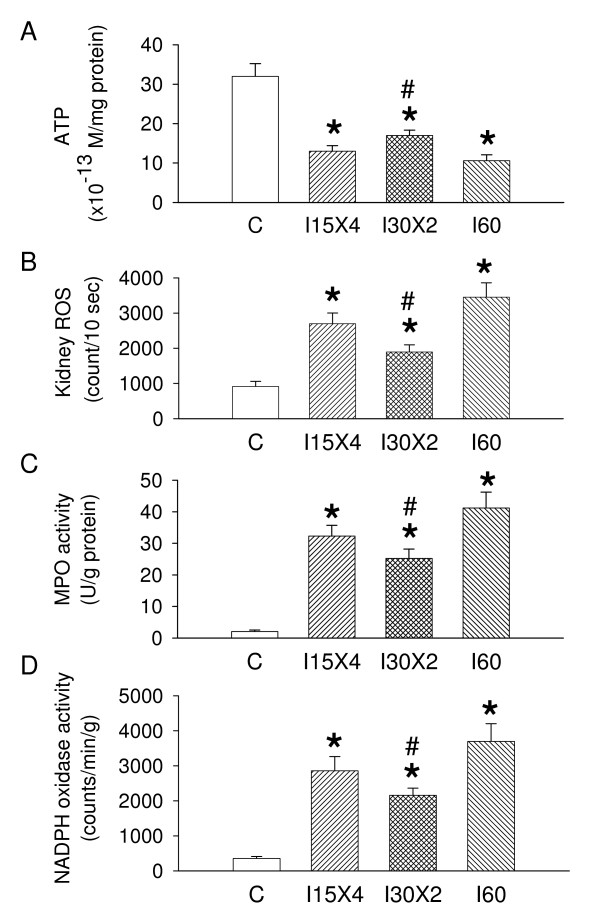
**Effect of ischemic conditioning on ATP content, reactive oxygen species (ROS), myeloperoxidase (MPO) and NADPH oxidase activity detection from the rat kidney**. A reduction in renal ATP content is noted in I15 × 4, I30 × 2 and I60 groups when compared to sham control group. The enhanced ROS (B), MPO (C) and NADPH oxidase activity (D) was significantly increased in the ischemia/reperfusion kidney with different ischemic conditioning treatment. The enhanced renal ROS amounts, MPO activity and NADPH oxidase activity are displayed in an order of I60 > I15 × 4 > I30 × 2 > sham control (C). Data are expressed as mean ± SEM (n = 6 in each group). *, *P *< 0.05 vs. C group. #, *P *< 0.05 vs. I60 group.

We measured ATP content in the I15 × 4, I30 × 2, I60 and sham control rats. We found that a reduction in renal ATP concentration was found in an order of I60 > I15 × 4 > I30 × 2 > sham control (Figure 2A). We determined the IC effect on O_2_^-. ^levels in the kidney subjected to IR injury. As shown in Figure 2B, the sham control kidney displayed a basal O_2_^-. ^level (914 ± counts/10 s). In I60 kidneys, increased O_2_^-. ^activity (3454 ± 407 counts/10 s) was found after 4 hours of reperfusion period. In the I15 × 4 kidneys, the level of O_2_^-. ^in reperfusion stages were 2898 ± 303 ocunts/10 sec. In the I30 × 2 kidneys, the level of O_2_^-. ^in reperfusion stages were 1897 ± 203 ocunts/10 sec. I30 × 2 treatment significantly depressed 40–50% of O_2_^-. ^level in the IR kidney. The enhanced renal MPO activity (Figure 2C) and NADPH oxidase activity (Figure 2D) are also consistent with the renal ROS data and displayed in an order of I60 > I15 × 4 > I30 × 2 > sham control.

### In situ localization of 4-HNE formation, tubular autophagy and apoptosis

**Figure 3:**
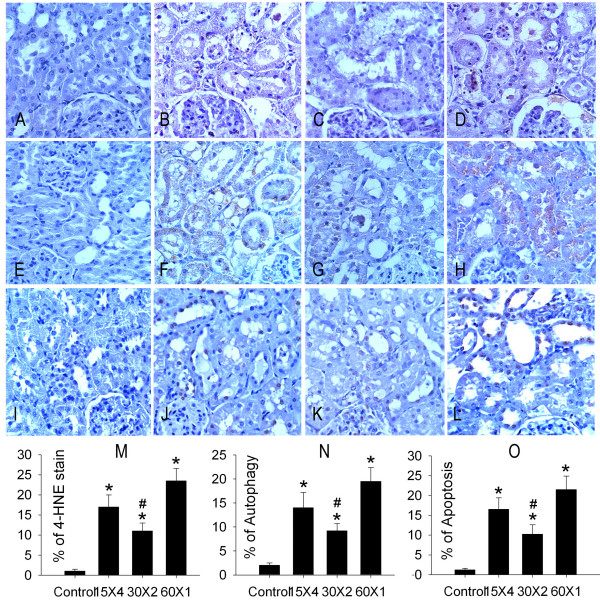
**Effect of ischemic conditioning on oxidative stress, autophagy and apoptosis in the kidney**. 4-hydroxynonenal (4-HNE, A-D) immunostaining of rat kidney sections. The kidneys with different treatment were immunostained with anti-4-HNE antibody. There is no 4-HNE immunoreactivity (brown color) present in the sham control kidney section (A). The 4-HNE immunoreactivity is present in both proximal and distal tubules of the 15 × 4 (B) and 60 × 1 (D) kidney. A less 4-HNE immunoreactivity is indicated in the 30 × 2 kidney (C). There is little stain of autophagy (E) and no apoptosis formation (I) in the sham control kidney. In response to I15 × 4, I30 × 2 or I60 × 1 treatment, all renal proximal and distal tubular autophagy (brown color, F-H) and apoptosis (brownish nuclei, J-L) are displayed in the insulted kidney. However, the mean data of 4-HNE stain (M), autophagy formation (N) and apoptosis production (O) were displayed in an order of 60 × 1 > 15 × 4 > 30 × 2 > sham control. * *P *< 0.05 vs. sham control (control) kidney. # *P *< 0.05 vs. 60 × 1 group.

We considered that the high levels of ROS might promote accumulation of renal oxidized products and contribute to autophagy and apoptosis. Renal tubular 4-HNE adducts were pronounced in the renal tubules of IR kidneys in an order of I60 (23.5 ± 3.1%) > I15 × 4 (17.0 ± 3.0%) > 130 × 2 (11.0 ± 2.0%) > sham control kidneys (1.0 ± 0.5%) (Figure 3A–D).

Apoptotic tubular cells were rarely detected in sections of sham control kidney (1.2 ± 0.4%) (Figure 3E). Renal tubular apoptosis were pronounced in the renal tubules of IR kidneys in an order of I60 (21.5 ± 3.4%) > I15 × 4 (16.5 ± 2.8%) > 130 × 2 (10.2 ± 2.4%) > sham control kidneys (1.2 ± 0.4%) (Figures 3I–L).

**Figure 4:**
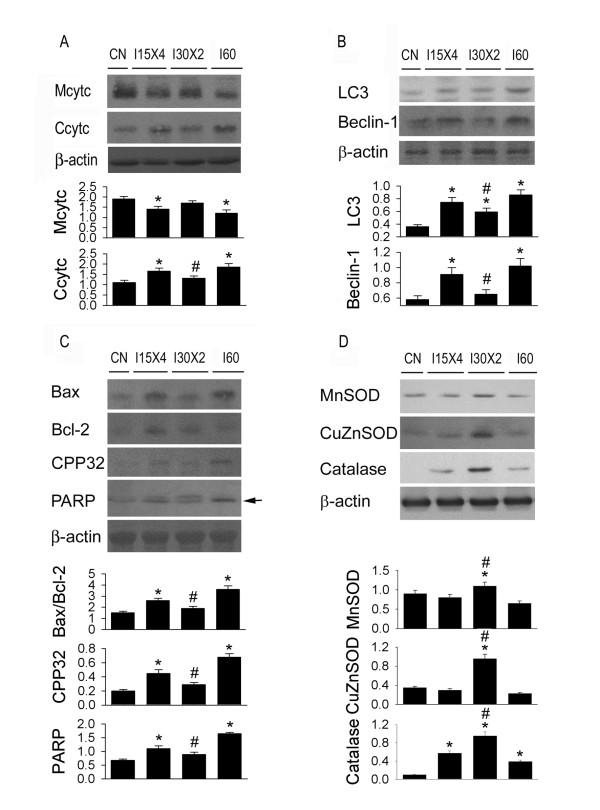
**Effect of ischemic conditioning on mitochondrial cytochrome C (Mcytc) and cytosolic cytochrome C (Ccytc) (A), autophagy- (B), apoptosis- (C), and antioxidant-related (D) proteins in the rat kidney subjected to ischemia/reperfusion injury**. I30 × 2 decreased Ccytc release when compared to I15 × 4 and I60 kidneys. In the autophagy-related protein expression, the expression in LC3 and Beclin-1 protein is implicated in an order of I30 × 2 > I15 × 4 > I60 > CN. In the proapoptotic mechanisms, increased Bax/Bcl-2 ratio, active CPP32 and PARP degradation expression are displayed in an order of I30 × 2 > I15 × 4 > I60 > CN. In the antioxidant protein expression, the expression in MnSOD, CuZnSOD amd catalase is demonstrated in an order of I30 × 2 > I15 × 4 > I60 > control (CN). *, *P *< 0.05 vs. CN group. #, *P *< 0.05 vs. I60 group.

In the basal level, Beclin-1 stain was rarely detected in sham-control kidney (2.0 ± 0.5%)(Figures 4E&4K). I60 (19.5 ± 2.9%), I15 × 4 (14.0 ± 3.2%) and I30 × 2 (9.2 ± 1.5%) treatment significantly increased (*P *< 0.05) renal tubular Beclin-1 stain. The mean data of 4-HNE (Figure 3M), autophagy (Figure 3N) and apoptosis (Figure 3O) is displayed.

### IC enhanced antioxidant-related protein and reduced apoptosis- and autophagy-related proteins expression

We explored mitochondrial and cytosolic cytochrome C (Figure 4A), LC3 and Beclin-1 expression (Figure 4B), Bax, Bcl-2, Bax, active CPP32, PARP degradation fragments (Figure 4C), Mn SOD, CuZn SOD and catalase (Figure 4D) in four groups of kidney samples by western blot. I60 treatment increased the translocation of cytochrome C from mitochondria to cytoplasm, whereas I30 × 2 treatment inhibited the translocation of cytochrome C from mitochondria to cytoplasm. In the sham control kidney, Mn SOD and CuZn SOD, not catalase, are detected. I30 × 2 treatment enhanced Mn SOD and CuZn SOD expression, but I60 and I15 × 4 treatment decreased Mn SOD and CuZn SOD expression in the kidney. However, I60, I15 × 4 and I30 × 2 treatment increased catalase expression in an order of I30 × 2 > I15 × 4 > I60 kidneys. Bax, Bcl-2, active CPP32 and PARP degradation fragments were sparsely detected in the sham control kidney. I60 markedly increased Bax, CPP32, and PARP expression, but did not affect Bcl-2 expression in the kidney. In contrast, I15 × 4 and I30 × 2 mildly enhanced Bax, Bcl-2, active CPP32, and PARP degradation expression in the kidney. I30 × 2 displayed a less Bax stain than that of I15 × 4 treatment. Beclin-1 and LC3 can be detected in the sham control kidney (Figure 4C). I60, I15 × 4 and I30 × 2 treatment increased LC3 and Beclin-1 expression in the kidneys. However, the expression was implicated in an order of I60 > I15 × 4 > I30 × 2 > sham control.

### IC ameliorated IR-induced renal dysfunction

**Figure 5:**
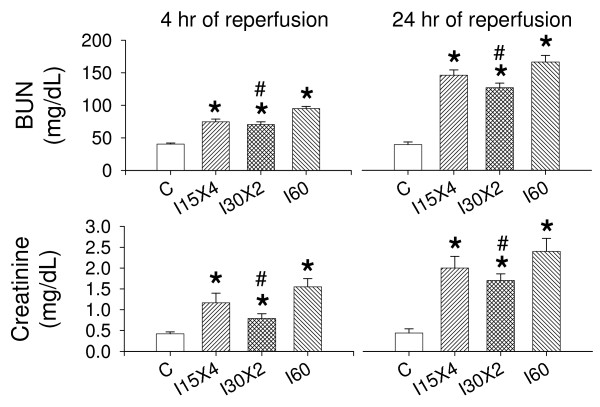
**Effect of ischemic conditioning on blood urea nitrogen (BUN) and plasma creatinine (Creatinine) in the kidney with ischemia/reperfusion injury**. In the I30 × 2 group (n = 6), BUN and Creatinine levels at 4 hours and 24 hours of reperfusion were significantly lower than those of I60 group. BUN and Creatinine levels in I15 × 4 treatment were not significantly different from I30 × 2 (n = 6) and I60 (n = 6) groups. Data are expressed as mean ± standard error mean. *, *P *< 0.05 vs. C group. #, *P *< 0.05 vs. I60 group.

As shown in Figure 5, after 4 hours of reperfusion, increased BUN and plasma creatinine levels were noted in I60, I15 × 4 and I30 × 2 kidneys. I30 × 2 treatment significantly decreased the values of BUN and creatinine when compared to the I60 group. I30 × 2 was more efficient than I15 × 4 treatment in reduction of BUN and creatinine than after 4 hours of reperfusion. Urine flow dramatically increased from 13.5 ± 2.0 μL/min (sham control) to 40.2 ± 4.0 μL/min (I15 × 4 group), 34.1 ± 3.2 μL/min (I30 × 2 group), and 47.5 ± 4.6 μL/min (I60 group) after 24 hours of reperfusion. Similarly, urine sodium excretion increased from 7.23 ± 1.1 μl/min/g in the control kidney to 16.5 ± 2.2 μl/min/g in I60 kidneys, 14.3 ± 2.0 μl/min/g in I15 × 4 kidneys and 12.3 ± 1.4 μl/min/g in I30 × 2 kidneys. The increased level of the sodium excretion was indicated in an order of I60 > I15 × 4 > I30 × 2 > sham control.

## Discussion

Our previous study found that preconditioning with hypoxia or adenoviral bcl-2/bcl-xL genes transfer to the kidney renders renal potential against IR injury including the reduction of oxidative stress, apoptosis and autophagy [20,21]. In this study, we demonstrated that with different IC condition during the ischemic period affords protective effect against IR induced renal oxidative injury.

During ischemia the cellular internal milieu resulting from hypoxia alone changes profoundly with the decrease in the ATP content, and increase in adenosine [7-9], intracellular accumulation of protons (a decreased pH level) [30], calcium [27] and hypoxanthine [31]. It has been suggested that enhanced breakdown of to adenosine and xanthine leads to increased production of O_2_^-. ^by xanthine oxidase that would cause functional and structural damage. O_2_^-. ^has been implicated in the natriuresis and the decrease in glomerular filtration and blood flow following ischemia/reperfusion in several reports [7,27,31]. These changes are complicated by the ROS stress during this stage of reperfusion [32]. Because of the adverse effect of ischemia and reperfusion, it is suggested that reduced IR damage can be improved by limiting ischemic injury during surgery or graft preservation or by protecting the organ from the aggression of the initial reperfusion with pharmacologic interventions [8,20,21,27]. The precise mechanisms of several preconditioning or postconditioning methods possibly involved the initial release of endogenous protective substances [7,9], which include adenosine, bradykinin, prostacyclin, nitric oxide, ROS as well as the Akt signaling transmitters [7,8,10,11]. Preconditioning protection can appear soon after sublethal ischemia/hypoxia and reappear 24–72 hours after ischemia/hypoxia [14,15,17]. Although preconditioning phenomena found soon after ischemia/hypoxia is not always related to de novo synthesis of proteins, ischemic preconditioning increases mitochondrial Mn SOD activity and reduces cytosolic cytochrome C release induced by IR [33]. Postconditioning treatment can trigger NO release and ameliorated tissue injury [7]. Our present study provided that a preserved ATP level was primarily found in the I30 × 2 group other than the I60 and I15 × 4 groups. We suggest that a lower level of adenosine accumulation may lead to a less production of ROS in the ischemic kidneys. The current data also demonstrated that IC with a 3-min reperfusion introduction between two stages of 30-min renal ischemia provides a significant renal protection by the reduction of renal ROS production, MPO and NADPH oxidase activity. The strong increase in urine flow and sodium excretion accompanied with increased in BUN and creatinine that we observed after 24 hours after ischemia/reperfusion injury, showing that ischemia/reperfusion induced O_2_^-. ^is able to induced renal tunular dysfunction. However, we found that ischemic conditioning with I30 × 2 is better than I60 and I15 × 4 in preservation of renal sodium excretory function and attenuation the increased level of BUN and creatinine.

Mitochondria are the target and source of ROS [34], which play an important role in physiologic signaling mechanisms and in regulation of apoptosis and autophagy pathways [19,20,35]. Mitochondrial dysfunction caused by inappropriate mitochondria permeability transition pore opening disrupts mitochondrial membrane potential for ATP synthesis and triggers oxidative and anoxic cell death [36]. The mitochondrial voltage-dependent anion conductance (VDAC) channel is responsible for cytochrome C release and is regulated by ROS and Bcl-2 family [37,38]. Bax can open it [24,39], and Bcl-2 and Bcl-xL stabilize and inhibit its opening [40]. Release of cytochrome C is a proximate trigger for evoking caspase 3 mediated apoptosis [38,39]. Our previous data showed that Bax and O_2_^-. ^are coexpressed in the IR kidney [21]. Increased ROS enhances Bax/Bcl-2 ratio, VDAC channel opening, cytochrome C release, and active caspase 3 mediated renal tubular apoptosis and Beclin-1/LC3 mediated autophagy [20,35]. In this study and our previous study [20], ischemia/reperfusion injury enhanced both apoptosis and autophagy production in the rat kidney and contributed to renal dysfunction. In the ischemia/reperfusion model, autophagy and apoptosis are concomitantly expressed in the rat kidney. IR enhanced Beclin-1 expression in the proximal and distal tubules by immunohistochemistry and increased LC3 and Beclin-1 expression in the IR kidney by Western blot [20], leading to renal dysfunction. Downregulation of Bcl-xL expression contribute to the apoptosis and autophagy after IR injury [20]. Bcl-xL inhibition by a human homologue of the Drosophila spin gene product (HSpin1) resulted in a caspase-independent autophagy cell death [41]. Overexpression of Bcl-xL inhibited the HSpin1-induced autophagy cell death [20,41]. Bcl-2 functioning as an antioxidant may prevent oxidant-induced cell death by increasing the capacity of mitochondria to store Ca^2+ ^[42]. Overexpression of Bcl-2 and Bcl-xL have recently been reported to inhibit ROS production, cytosolic cytochrome c release, active CPP32 and PARP degradation-mediated apoptosis and Beclin-1/LC3 mediated autophagy [20,21,43]. Increased Mn SOD mRNA levels after oxidative stress is reported to induce mitochondrial protection repair or turnover [44]. Catalase can inhibit autophagy by decreasing ROS accumulation and cell death [45]. Increased SOD and catalase activity decreased autophagosomes and mitochondrial damage in the pancreatic acinar cells [46]. In the present study, IC with one period of 3-min reperfusion between two 30-min ischemic periods can reduce oxidative stress, cytosolic cytochrome C release, apoptosis and autophagy. The mechanisms are due to the upregulation of several antioxidant proteins and downregulation in Bax/Bcl-2 ratio, active CPP32, PARP degradation and LC3 and Beclin-1 protein expression in the kidney.

In conclusion, IR injury is clinically relevant during renal surgical procedures such as anatrophic nephrolithotomy and kidney transplantation. The improvement of surgical approach by ischemic conditioning from the current result shows that during 60 min of renal ischemia, one interruption of 3-min reperfusion between two stages of 30-min renal protects the kidney from subsequent ischemia/reperfusion injury by the reduction of oxidative stress and mitochondrial dysfunction and subsequently reducing oxidative stress induced apoptosis and autophagy and renal dysfunction.

## Competing interests

The authors declare that they have no competing interests.

## Authors' contributions

CCT, WHH, HTY and LMK conceived the hypothesis. CCT conducted the statistical analyses for this manuscript. WHH, HTY, CCT and LMK drafted the manuscript. WHH, CCT, HTY, and LMK contributed to the design and conduction of the study. All the authors critically revised the drafted manuscript.
